# Perspectives of Parents and Health Care Providers about (Non)Medical Treatment in Infants with Reflux

**DOI:** 10.3390/pharmacy8040226

**Published:** 2020-11-23

**Authors:** Santina Gorsen, Koen Boussery, Myriam Van Winckel, Rolinde Demeyer, Eline Tommelein

**Affiliations:** 1 Department Pharmaceutical Sciences (FARM), Faculty of Medicine and Pharmacy, Vrije Universiteit Brussel, Laarbeeklaan 103, 1090 Jette, Belgium; 2 Pharmaceutical Care Unit, Faculty of Pharmaceutical Sciences, Ghent University, 9000 Ghent, Belgium; Koen.boussery@ugent.be; 3 Department of Pediatrics and Medical Genetics, Faculty of Medicine and Health Sciences, Ghent University, 9000 Ghent, Belgium; Myriam.vanwinckel@uzgent.be; 4 Department Pharmaceutical Sciences (FARM)—Laboratory Pharmaceutical Chemistry, Drug Analysis and Drug Information (FASC), Faculty of Medicine and Pharmacy, Vrije Universiteit Brussel, Laarbeeklaan 103, 1090 Jette, Belgium; Rolinde@opdegroei.be (R.D.); eline.tommelein@vub.be (E.T.)

**Keywords:** antacid, patient perspectives, paediatrics, community pharmacist, general practitioner, patient-centred care

## Abstract

Background: Reflux occurs in 50% of healthy infants at some point. This is most often a physiological condition and does not require drug treatment. Various studies have shown that the use of drugs affecting gastric acidity (DAGAs) in infants is increasing. This entails disadvantages such as unnecessary exposure of infants to medication and their side effects and a higher cost to society. Objective: To get an image of the current practice in Flanders regarding diagnosis and treatment of gastro-oesophageal reflux disease (GORD) in infants and the associated use of DAGAs. To this end, we determined both parents’ and health care providers’ experiences and perceptions about these treatments. Method: An observational cross-sectional study was conducted in April and May 2019. We developed a questionnaire for parents and three different questionnaires for health care providers (HCPs), including midwives, general practitioners, paediatricians and community pharmacists (CPs). The questionnaire for parents was only available through an online platform. HCPs were questioned face-to-face and through an online platform. Results: This study made clear that the counselling of children with GORD is multidisciplinary as the median number of counselling HCPs is 3 (interquartile range (IQR) = 2–4). 63% of the included 251 parents also seek support through online forums and groups. 60% of parents report that no physical tests were performed before DAGAs were prescribed and 39% of parents additionally state they perceived no effect of the prescribed DAGAs. Although parents reported to understand HCPs well (average score 7.4/10), satisfaction with care and information provision was scored lower (between 4.8 and 6.1/10). On the other hand, 234 HCPs answered the questionnaire, of which 89 midwives, 78 community pharmacists and 67 physicians. Only 45 HCPs indicate that guidelines to diagnose or treat GORD are clear. Physicians confirm they perform very little physical testing before starting DAGAs. Provided nonmedical measures to patients are largely in line with the European guidelines, however perceived effectiveness is moderate. Conclusion: Parents are in need for more information about tests, nutrition and (non)medical measures. HCPs on the other hand are in need for clear guidelines on diagnosing and treating GORD.

What is known? GORD *symptoms* in infants are prevalent, however significantly declining with increasing age. The use of drugs affecting gastric acidity (DAGAs) in infants is increasing, although there is no reason to believe the incidence of GORD is on the rise. 

What is new? Parents state they understand HCPs well. Nevertheless, perceived satisfaction with care and information provision is rather low. Parents mainly find support through online channels. The majority of health care providers state no clear guidelines on diagnosing and treating GORD are available.

## 1. Introduction

Gastro-oesophageal reflux (GOR) is defined as the retrograde flow of gastric content into the oesophagus and is very common in the first year of life [1,2]. Reflux can be associated with regurgitation (passive expulsion) or vomiting (active expulsion) [3]. GOR is physiological and harmless in the majority of infants. In moderate to severe cases however, GOR might assume pathological characteristics, evolving to gastro-oesophageal reflux disease (GORD). GORD is diagnosed when reflux is associated with feeding problems, failure to thrive, esophagitis and/or lung aspiration [2]. Many symptoms are non-specific which makes diagnosing more difficult. A recent systematic review reported a pooled prevalence of GORD *symptoms* in infants of 26.9% (95% confidence interval [CI] 20.1–33.7) ranging from 23.1% to 40.0%. Studies reporting prevalence at different time points up to the age of 13 months, showed a significant declining with increasing age [1]. 

The management of GORD includes nonmedical measures, such as promoting the time the infant spends in the head-elevated prone position and avoiding cigarette smoke. Also dietary adjustments (lowering volume intake of feeds, cow milk free diet, increasing viscosity of milk, adding probiotics) are widely adopted conservative measures. The evidence for these measures is low, but as they involve little risk they continue to play a basic role in the treatment [3]. Dietary adjustments may nevertheless have disadvantages, such as a higher cost of formula or poorer nutrition.

Next to nonmedical measures, the use of drugs affecting gastric acidity (DAGAs) can be indicated in the treatment of GORD. We include neutralising agents (antacids), H_2_-anithistamines (H2A) and proton pump inhibitors (PPIs). These do not counteract the reflux, but neutralise or reduce the acid production in the stomach, rendering the reflux non-irritating to the oesophagus or the upper airway mucosa [3]. The use of DAGAs in infants is increasing, although there is no reason to believe the incidence of GORD is on the rise [4,5,6,7]. This could mean there is overprescribing of medication. An Australian study for example found the number of physiological reflux diagnoses to decrease and the number of GORD diagnoses to increase. In that study, more than 40% of physicians prescribed DAGAs for physiological reflux [6]. Additionally, a large study of US healthcare databases showed that from 1999 to 2004, there was a more than 7-fold increase in PPI prescriptions. One of the PPIs, that is available in a child-friendly liquid formulation, even saw a 16-fold increase [4].

As the infants receiving the medication are unable to make health-related decisions, it is the responsibility of the parents and health care providers (HCPs) to decide whether medication is started. The perspectives towards medication use by both parties are therefore a crucial influential parameter. Although patients’ perspectives are increasingly gaining importance, very little research considering this topic has been performed. Therefore, the goal of this study is to map the experiences and perspectives of both parents and HCPs regarding the diagnosis and treatment of GORD. 

## 2. Methods

### 2.1. Study Design and Setting

We conducted an observational, cross-sectional study in April and May 2019 in Flanders (the Dutch-speaking part of Belgium). In this study, we surveyed parents’ experiences and perspectives on the use of DAGAs through an online questionnaire. Additionally, general practitioners, paediatricians, community pharmacists and midwives were questioned about GORD in infants and the associated use of DAGAs. This could be online or face-to-face. The study was set up by pharmacists and a paediatrician. The study was approved by an independent Committee for Medical Ethics affiliated with UZ Gent (registration number: B670201939986). All participants provided consent upon starting the questionnaire.

### 2.2. Participants

Every parent in Flanders with at least one child aged 6 years or less, who took an antacid in the first year of life was eligible for inclusion. All respondents had to speak Dutch and voluntarily agree to fill in the questionnaire. The goal was to reach at least 200 parents. Sample sizing was based on feasibility rather than calculations. Respondents were recruited through convenience sampling and online sampling, such as nurseries and social media. A list with the largest cities by province was drawn up. In these cities, all nurseries were contacted by phone or email. All nurseries that agreed to participate were asked to disseminate the questionnaire.

The inclusion criteria for the HCPs were dependent on their function. Physicians were eligible to complete the questionnaire if they had already treated, diagnosed, or followed up at least ten infants with GORD. Community pharmacists were eligible if they had dispensed Gaviscon^®^ Baby, omeprazole and/or ranitidine for infants within the preceding year. The inclusion criterion for midwives involved at least one experience with an infant with GORD. All respondents had to speak Dutch. The goal was to reach a total of 150 healthcare providers, via online questionnaires. Here as well, sample sizing was based on feasibility rather than calculations. Questionnaires were disseminated through the newsletter of the respective professional organisations. All questionnaires were as well distributed through social media to reach a wider audience.

### 2.3. Questionnaire and Outcomes

#### 2.3.1. Parents’ Survey

The parents’ survey was divided into different sections: (1) demographics, (2) experiences and perspectives about GORD treatment and prescribed medication and (3) infant nutrition. The latter assessed food habits considering breastfeeding, bottle feeding and solid nutrition, including the use of food thickeners.

#### 2.3.2. Healthcare Providers’ Surveys

Three different questionnaires were drawn up: one for physicians (both general practitioners and paediatricians), one for community pharmacists and one for midwives.

The midwives questionnaire was divided into three sections: (1) demographics, (2) recognition of GORD in infants and (3) treatment of GORD in infants. The section ‘Recognising GORD in Infants’ asked how the midwife recognises GORD and when they refer to a physician. The section ‘Treatment of GORD in Infants’ questioned how often nonmedical advice was provided. Perceived effectiveness and safety of nonmedical and drug treatments was questioned.

The questionnaire for the community pharmacists was divided into three sections: (1) demographics, (2) dispensation protocol and (3) compounding. The ‘dispensation protocol’ section asked about the dispensation protocol for DAGAs and used information sources. They were also asked whether interactions between infant formula and DAGAs are known and what drug advice they give to parents with children with GORD. Possible interactions between casein-based formulas and DAGAs may be taken into account because of its possible loss of thickening properties due to neutralisation of the acidic environment. The final section, ‘Compounding’, questioned which guidelines are used for the compounding of baby-friendly omeprazole and ranitidine formulations.

The physician questionnaire was divided into three sections: (1) demographics, (2) diagnosis of GORD in infants and (3) treatment of GORD in infants. The section ‘Diagnosis of GORD in Infants’ examined which symptoms the physician correlates with GORD and which tests are performed to make a diagnosis. The section ‘Treatment of GORD in Infants’ questioned how often the physician provides nonmedical advice to parents and initiates certain drug treatments. Both perceived effectiveness and safety of drug treatments were questioned.

### 2.4. Statistical Analysis

Results were processed using Excel^®^ 2016 (Microsoft Corporation, Redmond, WA, USA) and SPSS statistics 25 (IBM Corp., Armonk, NY, USA) were used. First, the answers were coded in Excel^®^ 2016. Descriptive statistics were used for the calculation of numbers with percentages, medians with interquartile range and means with standard deviations as appropriate. Independent samples T-tests were performed to compare the means.

## 3. Results

### 3.1. Parents’ Survey: Demographics, Perceptions and Perspectives

Total of 251 parents completed the questionnaire. Demographic data and reported symptoms and pathologies of children are shown in Table 1.

#### 3.1.1. Experiences and Perspectives about Treatment

The first healthcare provider with whom parents came into contact with was in 105 cases (42%) a paediatrician, in 64 cases (25%) a midwife and in 50 cases (20%) a general practitioner. The remaining were osteopaths (14 cases–6%), governmental nurses/physicians (8 cases–3%), pharmacists (4 cases–2%), homeopaths (1 case) or a lactation consultant (2 cases). Parents reported to be counselled by a median of three different HCPs (IQR: 2–4). Total of 230 parents (92%) were counselled by a paediatrician, 157 (63%) by an osteopath, 125 (50%) by a general practitioner, 104 (51%) by a midwife, 99 (39%) by a pharmacist and 50 (20%) by a dietician. Many other HCPs were mentioned, such as lactation consultants, homeopaths, kinesiologists, foot reflexologists, but never by more than five parents. Besides HCPs, parents sought support in online forums or support groups (n = 159, 63%), with family (n = 135, 54%), with friends (n = 129, 51%) and websites (n = 141, 56%). About 16 parents (6%) sought help through live support groups. 

Total of 151 parents (60%) reported that no physical tests were performed before DAGAs were prescribed. About 70 children had one test, 20 children had two tests and 10 had more than two. Most performed tests were 24 h PHmetry (58 children), endoscopy (21 children), stomach emptying study (11 children) and Barium contrast radiography (10 children). All other tests were performed in less than 10 cases. 

On a scale from of 0 (=no understanding) to 10 (=full understanding), they scored the information by all HCPs between 7.2 and 7.5 out of 10 with a standard deviation of 2.7 to 2.9. Considering satisfaction with the physical tests, parents were less satisfied. On a scale from 0 (=no satisfaction) to 10 (=full satisfaction), they scored a mean of 4.8 (standard deviation (SD) 3.0) and 83 parents (33%) would have wanted more information about the tests. Satisfaction with advice around feeding was scored 5.8 (SD 3.0) but 114 parents (45%) still would have liked more information about this topic. Satisfaction with the treatment in general was scored 5.9 (SD 2.8) and satisfaction with the HCP 6.1 (SD 2.9). However, 61 parents (24%) wanted to have received more information about the pharmacotherapy and 88 parents (35%) wanted to have received more information about the nonmedical therapy.

#### 3.1.2. Experiences and Perspectives about Prescription Medication

Total of 72 parents (29%) reported they would accept any treatment to improve the health of their child. About 242 (96%) parents indicated they would like to participate in the decision to choose a treatment for their child. Total of 235 (94%) stated they would choose the treatment based on the pros and cons that a HCP discussed with them. 

Of the 251 included parents, 107 (43%) reported that at one point, their child was prescribed neutralizing agents, 110 (44%) said their child was prescribed an H2A and 188 (75%) said their child was prescribed a PPI. About 33 parents (13%) reported that most medications were difficult to understand. Total of 40 parents (16%) were constantly afraid they would forget to give the medication to their child and 56 parents (22%) reported they sometimes forgot to take the medication when they left the house. About 94% (237 respondents) believes the medication is used to keep the symptoms under control. However, 99 parents, (39%) perceived that the medication did not help at all.

#### 3.1.3. Nutrition

At one point 145 children (58%) received *breastmilk* during the treatment with DAGAs. About 34 of them (23%) received thickened breastmilk. Used thickeners were carob bean gum (23 cases), sodium carboxymethylcellulose (NaCMC–12 cases) and starch (6 cases), the latter is predominantly based on rice. Total of 183 (73%) of children received *infant formula* at one point during the treatment with DAGAs. Moreover, 55 children (30%) were at some point advised to use specific ant reflux formula, 105 were at some point advised to use formula based on extensive protein hydrolysates (57%) and 65 were prescribed an infant formula based on amino acids (36%). Around 119 parents (65%) bought their formula from the pharmacy.

Total of 178 children (71%) received *solid foods* during the treatment with DAGAs. Children were on average 4.8 months old (SD 1.23) when solids were introduced. Twenty-seven parents (15%) introduced solids through baby led weaning, 139 (78%) started with pureed foods. The remaining 12 (7%) were introduced through a mix of both methods. Forty-eight parents (19%) thickened purees, of which 46 used a starch-based thickener, 1 carob bean gum and 1 NaCMC. 

### 3.2. Health Care Providers’ Surveys

#### 3.2.1. Demographics

In total, 234 HCPs answered the questionnaire, of which 89 midwives, 78 community pharmacists and 67 physicians. The demographic data are presented in Table 2.

#### 3.2.2. Diagnosis and Treatment of GORD

Total of 11 midwives (MW) (12%) and 34 physicians (Ph) (51%) think there are clear guidelines on recognizing or diagnosing GORD. Only 6 midwives (7%) think there are clear guidelines on treating GORD, compared to 30 physicians (45%). The following percentage of HCPs associated the subsequent symptoms with GORD: postprandial regurgitation (MW: 61%–Ph: 37%), postprandial vomiting (MW: 58%–Ph: 31%), arched back crying (MW: 85%–Ph: 63%), difficulties feeding (MW: 60%–Ph: 72%), crying after feeding (MW: 87%–Ph: 72%), chronic cough (MW: 43%–Ph: 75%), oesophagitis (MW: 66%–Ph: 91%), bad breath (MW: 51%–Ph: 21%), failure to thrive (MW: 35%–Ph: 58%), inadequate weight gain (MW: 60%–Ph: 81%), breathing difficulties (MW: 26%–Ph: 28%), recurrent pneumonia (MW: 28%–Ph: 58%) and oesophageal stricture (MW: 9%–Ph: 28%).

Considering the last 10 patients diagnosed with GORD, sixty percent (40/67) of the participating physicians did not use a standard questionnaire to evaluate the symptoms. Fifty-four physicians (81%) declared to have included a general physical examination, however, only 6 physicians used a pHmetry (9%), 11 used combined multiple intraluminal impedance (MII) (16%), 8 used barium contrast radiography (12%), 15 used an upper gastric-oesophageal endoscopy with biopsy (22%) and 5 used a gastric emptying study (8%). Total of 97% (65) of the participating physicians state that from time to time, they doubt their diagnosis of GORD. Considering the last 10 patients diagnosed with GORD, 31% (21/67) of the physicians consulted with or referred to another paediatrician in at least one case. From all participating physicians, 40% (27/67) prescribes DAGAs immediately after diagnosing the infant with GORD.

#### 3.2.3. Nonmedical and Medical Advice

Only 19% of midwives (17/89) would advise the use of neutralizing agents such as sodium bicarbonate without first referring to a physician. Twenty-one midwives (24%) mention they also advice ‘Other products or procedures’. These include probiotics (13 mentions), osteopathy (5 mentions), fennel drops (4 mentions) and ginger (3 mentions). Simethicone was mentioned once. About 79% of midwives often inform themselves about the use of DAGAs in infants. Most used sources include continued education classes (55%), the Belgian Centre for Pharmacotherapeutical Information (47%) [8], the Summary of Product Characteristics (35%), scientific reports and reviews (24%) and the Pharmacotherapeutical Compass [9] (9%) (see Table 3 and Table 4).

Of the interviewed community pharmacists (CPs), 13% reported to have access to a dispensing protocol. About 69% (88/78) of the pharmacists search for more information about the prescribed medication when dispensing them. None of the CPs provide information leaflets about the dispensed DAGA or the underlying diagnosis. Around 5% (4/78) of the pharmacists refer to an informational website. Nevertheless, all pharmacists stated to provide nonmedical advices such as the position of the bed, adding thickeners to the normal diet, changing the soother and considering changing the normal diet.

Also 15% of the physicians mention they sometimes recommend ‘other products or procedures’, such as probiotics, ginger- or fennel-based products, simethicone and domperidone. Of all physicians, 48% answered to sometimes feel a particular pressure from parents to prescribe. The other half (51%) answered to (almost) never feel such pressure. About 37% of the physicians state the risk of adverse events influences their prescribing behaviour a lot, 45% state that it has a medium influence, 12% say it has almost no influence and 6% say it has no influence. Not all physicians are aware of the possible side effects of DAGAs. Total of 57% of the physicians report on constipation during the use of Gaviscon baby^®^ and 18% state cramps as a possible side-effect. 42% of the physicians mention an increased risk of infections and 28% of headaches because of the use of PPIs.

#### 3.2.4. Interaction with Normal Diet

About 52% of midwives (46/89) take the diet of the child into account when counselling the intake of DAGAs. All of them specifically consider thickened formulas. Among pharmacists, 55% (43/78) check the child’s diet when dispensing DAGAs and 56% (44/78) believe that DAGAs can interact with a normal diet (=the milk diet). When prescribing medication, 31% (21/67) of the physicians take possible interactions with the child’s diet into account.

## 4. Discussion

### 4.1. Main Findings of This Study

With the growing importance for patient-centred care, we aimed to evaluate the experiences and perceptions of parents towards the use of DAGAs in their children. The perspectives of HCPs towards these medications were studied in parallel. This study made clear that the counselling of children with GORD is multidisciplinary. Parents mainly seek support through online forums and groups. Both parents and physicians state that—before starting DAGAs—none or only few physical tests are performed. While 75% of the parents stated that their infant was prescribed a PPI, a significant proportion of parents additionally state they perceived no effect of the prescribed DAGAs. Although parents state they understand HCPs well, perceived satisfaction with care and information provision is rather low. Midwives estimated the safety of neutralizing agents, H2A and PPIs is lower than physicians. The majority of midwives and physicians indicate that guidelines to diagnose or treat GORD are unclear. 

### 4.2. What Is Already Known about This Subject

In an observational study in 64 infants with persistent reflux symptoms, 90% of included infants were already on DAGAs at the start of the study. A pHmetry was performed on 44 of the 64 children, showing only eight infants with an abnormal result. In the remaining 51 infants, DAGAs were discontinued and the parents were advised about the position of the child and about reduced food intake, possibly combined with smaller portion sizes. In 87% of these infants who were followed up, there was no worsening of symptoms, sometimes even an improvement [10]. The authors conclude there was overprescribing of DAGAs as the majority of infants did not meet the diagnostic criteria for GORD. This could be due to the lack of evidence for GORD as a pathology among infants. Although we did not perform diagnostic testing, we believe that overprescribing could also be the case in our study. There are barely physical tests performed and almost forty percent of parents state the antacid medication didn’t have any effect. Another prospective observational study from Australia questioning 400 physicians reported that ninety percent indicated to empirically prescribe medication to infants as a diagnostic method [11]. However, this is discouraged by the ESPGHAN guidelines. This is further motivated by a randomised controlled trial where no difference in GORD symptoms was found between treatment with a PPI and a placebo [12], rendering it a useless screening method.

In the study by Khoshoo, 80% of the infants received thickened food, but in 70% the food appeared to be insufficiently thickened (i.e., less than prescribed concentrations). Also, 40% of the infants had an excessively high energy intake [10]. Use of thickening agents is also prevalent in the current study. We did not evaluate the doses, but specifically the thickening of solid foods increases the risk for excessive energy, intake, as in our study almost all parents used starch-based thickeners to thicken pureed foods. However, physicians and midwives mostly or sometimes advise no-calorie thickeners and never or rarely add calorie-rich thickeners such as starch. Although no evidence for thickening mother’s milk for GORD symptoms is available, North American Society for Paediatric Gastroenterology, Hepatology, and Nutrition (NASPGHAN) and the European Society for Paediatric Gastroenterology, Hepatology, and Nutrition (ESPGHAN) guidelines included this strategy in their recommendations, taking caution in consideration [3,13]. In this study, almost a fourth of included parents reported to have applied this measure. As well, we observe that—although incompatible, some patients still thicken breastmilk with starch-based thickeners [3]. 

### 4.3. What This Study Adds

This study gives an elaborate view on the experiences and perceptions of parents about treating their infant with DAGAs. Almost all parents wanted to be involved in their infant’s therapy. This is in line with previous research considering the involvement in the drug therapy for pain, inflammation of infections in infants where about 75% of parents indicated they find involvement ‘quite’ or ‘very’ important [14]. The abundant use of online forums or support groups and websites to find information and support, shows that HCPs should pay specific attention to refer people to evidence-based websites or forums with trustworthy information. This might be explained by the finding that parents understand HCPs well, although a significant amount felt not informed enough about physical tests, feeding or (non) medical approaches.

A large majority of midwives and physicians state that guidelines on recognising or diagnosing GORD are not clear. This might be the reason why almost all physicians state that from time to time, they doubt their diagnosis of GORD. Analogously, guidelines on treating GORD are also perceived as being unclear. This should be a wake-up call for organisations such as NASPHGAN/ESPGHAN to investigate why they need clarification and adapt them accordingly.

This is one of the few studies that has researched timing of the introduction of solid feeds. With a mean introduction age of 4.8 months (≈20.5 weeks), the WHO guideline of exclusive milk feeding up until 6 months of age is not reached. It does fall within the timeframe of 17 to 24 weeks, set by the ESPGHAN guideline [15]. However, while the early introduction of solid feeds appeared not protective to reflux in previous research [16], it could however undermine continued breastfeeding.

Considering the perspectives of HCPs, it is clear that HCPs feel uncertain about diagnosing or treating GORD. The recommended nonmedical measures are nevertheless much in line with the recommendations by the NASPHGAN/ESPGHAN committee [3]. The only remark is that—although the use of complementary treatments such as prebiotics, probiotic, or herbal medications to treat GORD are not recommended—still some HCPs advise them.

Additionally, it is remarkable that midwives consistently perceive the safety of DAGAs lower than physicians. The results suggest that not all physicians are aware of the possible side effects, although constipation, cramps and a higher infection risk is known to be associated with the use of DAGAs. Although the use of PPIs and H2As is regarded as safe, the efficacy is rather poor [17,18].

Only 44% of the CPs deem to have adequate and sufficient information to provide to the parents regarding the safety of the use of medication. The CPs find their information in several scientific sources.

### 4.4. Strengths and Limitations

A strength of the study is that we included perceptions of multiple HCPs, as almost all parents indicate being counselled by various HCPs. Studying perception of parents and HCPs in parallel gave us the opportunity to evaluate how current practices are perceived by both parties. Furthermore, the study was conducted and reported according to the Strengthening the Reporting of Observational Studies in Epidemiology (STROBE) guidelines. 

Nevertheless, this study has some limitations. First, the parents may have responded differently when participating in research. Social desirability bias can therefore not be excluded and may lead to a more positive presentation of the results. However, by guaranteeing anonymity of participants this was limited as much as possible. This is also reflected in the fact that the results of our study are comparable to those of previous studies. The next and largest limitation is the low generalisability. The method of recruitment implicates that mainly motivated or interested parents and HCPs took part in this study, leading to a participation bias. Indeed, almost a quarter of the participating parents work in the healthcare sector, compared to about 5% in the total Flemish population. Moreover, the majority of participating physicians were paediatricians. Additionally, the proportion of the highly educated in this study was fairly large (79%). Data from Statbel indicated that in 2016, 30% of the Belgian population finished higher education. It would be useful to carry out a study that includes more participants of different ethnic origins and with a better distribution in terms of educational attainment.

### 4.5. Implications for Future Research and Practice

First of all, clear definitions of GOR and GORD should be drawn up [19]. As well, adequately powered, randomised controlled trials should be used to investigate the efficacy of both nonmedical and medical interventions. We believe this can further improve the guidelines to treat GORD. 

From this research, it is clear that parents want to be involved in the decision whether to start antacid medication for their child, based on a discussion of the pros and cons with a HCP. Despite understanding HCPs sufficiently, parents perceived received care and information below par. For all questioned subjects (performed tests, nutrition, nonmedical measures, pharmacotherapy), between one in four and one in three parents wanted to have received more information. In this study, almost all children were counselled by a paediatrician. They would therefore be the key HCP to elaborate on these topics during the consultations or refer to other HCPs such as midwifes, pharmacists and dieticians for further counselling. In other countries however, this might not be the case and the key HCP might be from a different profession. Professional organisations might also consider being part of online support groups to finetune and double check the information. Adequate provision based on the parent’s need, should as well occur when dispensing DAGAs for infants.

Additionally, multidisciplinary courses on both diagnosing and treating GORD should be rolled out. This would make all HCPs more confident and would align the given advices. HCPs should also be aware of the existence of support groups and qualitative online documentation so they can refer parents to evidence-based information sources. Although it might be a challenge, since a good evidence base is lacking for diagnosis and treatment of GORD among infants.

## 5. Conclusions

This study made clear that the counselling of children with GORD is multidisciplinary. Parents mainly seek support through online forums and groups. Both parents and physicians state that—before starting DAGAs—none or only few physical tests are performed. A significant proportion of parents state additionally they perceived no effect of the prescribed DAGAs. Although parents state they understand HCPs well, perceived satisfaction with care and information provision is rather low. The majority of midwives and physicians indicate that guidelines to diagnose or treat GORD are unclear. However, the suggested nonmedical measures are in line with the recommendations. 

## Figures and Tables

**Table 1:** Demographic data of included parents and reported symptoms and pathologies of children. (n = 251).

Demographic Data of Included Parents and Reported Symptoms and Pathologies of Children	n = 251
**Female gender, n (%)**	241 (96)
**Age, median (IQR)**	32 (29–34,5)
**Origin, n (%)**	
Caucasian	243 (97)
Other or not specified	8 (3)
**Age of participants’ children, median (IQR)**	1 (2–4)
**Number of children per participant, median (IQR)**	2 (1–2)
**Education, n (%)**	
Primary education	2 (1)
Secondary education	51 (20)
Higher education	198 (79)
**Job sector, n (%)**	
Healthcare sector	60 (24)
Education	43 (17)
Government	11 (4)
Other	137 (55)
**Number of parents reporting this symptom present in their child before antacid was started, (%)**	
Arched back crying	212 (84)
Excessive crying	212 (84)
Sleeping disorders	161 (64)
Regurgitation	118 (47)
Refusing to eat	84 (33)
Vomiting	75 (30)
Projectile vomiting/Forceful vomiting	59 (24)
Weight loss	54 (22)
Breathing difficulties	32 (13)
**Number of parents reporting this underlying pathology(ies) for which DAGAs were prescribed, (%)**	
Only reflux	77 (31)
Reflux and CMPA	73 (29)
Reflux, GORD and CMPA	15 (6)
Only GORD	14 (6)
GORD and CMPA	14 (6)
Reflux and GORD	12 (5)
Only CMPA	6 (2)
Other (combinations of) indications *	40 (16)
Number of parents reporting their child received this/these medication(s), (%) **	
Only neutralizing agent (e.g., sodium bicarbonate)	17 (7)
Only H_2_-anthihistamine	34 (14)
Only PPI	76 (31)
Neutralizing agent + H_2_-anthihistamine	10 (4)
Neutralizing agent + PPI	46 (18)
H_2_-anthihistamine + PPI	32 (13)
Neutralizing agent + H_2_-anthihistamine + PPI	34 (14)

***CMPA*** : cow milk’s protein allergy; **GORD**: gastro-oesophageal reflux disease; **PPI**: proton pump inhibitor. * other (combinations of) indications included colic, oesophagitis, hidden reflux and problems with stomach emptying ** n = 249, 2 missing data.

**Table 2:** Demographic data of the included health care providers (HCPs): midwives, community pharmacists and physicians.

Demographic Data of Included HCPs: Midwives, Community Pharmacists and Physicians	
**Midwives (n = 89)**	
Female gender, n (%)	89 (100)
Age, median (IQR)	37 (29–47)
Number of midwives with (grand) children, n (%)	70 (79)
Number of midwives with (grand) children that used DAGAs in the first year of life, n (%)	23 (26)
Years of experience, median (IQR)	12 (4.75–25)
Number of midwives working in a group practice, n (%)	52 (58)
Number of families a month per midwife, median (IQR)	15 (10–31.25)
Infants counselled with physiological reflux in the past month, n (%)	
None	4 (4)
1–4	49 (55)
5–9	21 (24)
10–14	12 (3)
More than 15	3 (3)
Infants counselled with GORD in the past month, n (%)	
None	42 (47)
1–4	44 (49)
5–9	3 (3)
10–14	0 (0)
More than 15	0 (0)
Community pharmacists (CP) (n = 78)	
Female gender, n (%)	66 (84)
Age, median (IQR)	31 (27–42)
Number of CPs with (grand) children, n (%)	44 (56)
Number of CPs with (grand) children that used DAGAs in the first year of life, n (%)	17 (22)
Years of experience, median (IQR)	8 (4–18)
Number of infants for whom Gaviscon^®^ Baby was dispensed in the preceding year, n (%)	
None	14 (18)
1–4	44 (56)
5–9	11 (14)
10–14	4 (5)
More than 15	5 (6)
Number of infants for whom omeprazole suspension was compounded and dispensed in the preceding year, n (%)	
None	7 (9)
1–4	45 (58)
5–9	13 (17)
10–14	7 (9)
More than 15	6 (8)
Number of infants for whom ranitidine suspension was compounded and dispensed in the preceding year, n (%)	
None	46 (59)
1–4	27 (35)
5–9	3 (4)
10–14	0 (0)
More than 15	2 (2)
Number of infants for whom Zantac^®^ syrup was dispensed in the preceding year, n (%)	
None	34 (44)
1–4	39 (50)
5–9	4 (5)
10–14	1 (1)
More than 15	0 (0)
Physicians (n = 67)	
Specialty, n (%)	
Paediatrics	60 (90)
General practice	7 (10)
Female gender, n (%)	52 (78)
Age, median (IQR)	41 (34–48)
Number of physicians with (grand) children, n (%)	62 (92)
Number of physicians with (grand) children that used DAGAs in the first year of life, n (%)	20 (30)
Years of experience, median (IQR)	13 (6–20)
Infants diagnosed with GORD in the past month, n (%)	
None	10 (15)
1–4	41 (61)
5–9	8 (12)
10 or more	8 (12)

***CP:*** *community pharmacist; **FTE:** full-time equivalents; **GP:** general practitioner; **PTA:** pharmaceutical technical assistant.*

**Table 3:** Summary of frequency of advising nonmedical measures in gastro-oesophageal reflux disease (GORD), as well as perceived effectiveness.

	Frequency of Advising This Measure					Perceived Effectiveness of Measure				
	Always	Mostly	Sometimes	Rarely	Never	Very	Moderately	Low	Not	Not Experienced
Provide advice on the child’s posture DURING feeding *										
Midwives (n = 89)	**52 (58)**	21 (24)	13 (15)	3 (3)	0 (0)	28 (31)	**55 (62)**	5 (6)	0 (0)	1 (0)
Physicians (n = 67)	18 (27)	13 (19)	**20 (30)**	11 (16)	5 (7)	8 (12)	**29 (43)**	25 (37)	5 (7)	0 (0)
Provide advice on the child’s posture AFTER feeding *										
Midwives (n = 89)	**77 (87)**	10 (11)	1 (1)	1 (1)	0 (0)	**58 (65)**	30 (34)	1 (1)	0 (0)	0 (0)
Physicians (n = 67)	**37 (55)**	21 (31)	8 (12)	0 (0)	1 (1)	16 (24)	**41 (61)**	10 (15)	0 (0)	0 (0)
**Adjust maternal nutrition if breastfeeding**										
Midwives (n = 89)	12 (13)	13 (15)	**33 (37)**	20 (22)	11 (12)	8 (9)	**39 (44)**	21 (24)	13 (15)	8 (9)
Physicians (n = 67)	4 (6)	16 (24)	**19 (28)**	12 (18)	16 (24)	4 (6)	**23 (34)**	19 (28)	21 (31)	0 (0)
**Switching from breastfeeding to formula**										
Midwives (n = 89)	0 (0)	1 (1)	1 (1)	12 (13)	**75 (84)**	0 (0)	8 (9)	13 (15)	**40 (45)**	28 (31)
Physicians (n = 67)	0 (0)	0 (0)	4 (6)	24 (36)	**39 (58)**	1 (1)	5 (7)	19 (28)	**42 (63)**	0 (0)
Change infant formula * (trial with extensive hydrolysate or anti-reflux formula)										
Midwives (n = 89)	2 (2)	19 (21)	**38 (43)**	24 (27)	6 (7)	20 (22)	**42 (47)**	18 (20)	5 (6)	4 (4)
Physicians (n = 67)	2 (3)	**32 (48)**	27 (40)	5 (7)	1 (1)	6 (9)	**45 (67)**	15 (22)	1 (1)	0 (0)
Change feeding frequency and/or portion *										
Midwives (n = 89)	9 (10)	**38 (43)**	33 (37)	8 (9)	1 (1)	37 (42)	**44 (49)**	7 (8)	0 (0)	1 (1)
Physicians (n = 67)	5 (7)	**27 (40)**	22 (33)	9 (13)	4 (6)	8 (12)	**35 (52)**	30 (16)	4 (6)	0 (0)
Adding no-calorie thickeners (carob gum–NaCMC) to normal diet (milk/purees) *										
Midwives (n = 89)	4 (4)	20 (22)	**29 (33)**	22 (25)	14 (16)	25 (28)	**37 (42)**	14 (16)	5 (6)	8 (9)
Physicians (n = 67)	2 (3)	**26 (39)**	23 (34)	13 (19)	3 (4)	5 (7)	**42 (63)**	16 (24)	4 (6)	0 (0)
Adding calorie-rich thickeners (starch) to normal diet (milk/purees) *										
Midwives (n = 89)	0 (0)	1 (1)	7 (8)	11 (12)	**70 (79)**	3 (3)	15 (17)	14 (16)	17 (19)	**40 (45)**
Physicians (n = 67)	1 (1)	1 (1)	8 (12)	17 (25)	**40 (60)**	1 (1)	13 (19)	19 (28)	**34 (51)**	0 (0)

* Recommended by the North American Society for Paediatric Gastroenterology, Hepatology, and Nutrition and the European Society for Paediatric Gastroenterology, Hepatology, and Nutrition.

**Table 4:** Summary of frequency of advising medical measures in GORD, as well as perceived effectiveness and safety.

	Frequency of Advising/Prescribing This Measure (Past One Month)				Perceived Effectiveness of Measure					Perceived Safety of Measure				
	0	1–4	5–9	10 or More	Very	Moderately	Low	Not	Not Experienced	Very	Moderately	Low	Not	Not Experienced
**Use of neutralizing agents (e.g., medication with sodium bicarbonate)**														
Midwives (n = 89)	NA	NA	NA	NA	4 (4)	**37 (42)**	**37 (42)**	11 (12)	0(0)	6 (7)	10 (11)	**33 (37)**	30 (34)	10 (11)
Physicians (n = 67)	29 (43)	**35 (52)**	3 (4)	0 (0)	1 (1)	19 (28)	**32 (48)**	15 (22)	0 (0)	25 (37)	**36 (54)**	5 (7)	1 (1)	0 (0)
**Use of H_2_-antihistamines (compound preparation or Zantac^®^)**														
Midwives (n = 89)	NAP	NAP	NAP	NAP	35 (39)	**42(47)**	10 (11)	2 (2)	0 (0)	6 (7)	22 (25)	**38 (43)**	19 (21)	4 (4)
Physicians (n = 67)	**52 (78)**	9 (13)	6 (9)	0 (0)	9 (13)	**46 (69)**	11 (16)	1 (1)	0 (0)	17 (25)	**46 (69)**	4 (6)	0 (0)	0 (0)
**Use of PPI’s**														
Midwives (n = 89)	NAP	NAP	NAP	NAP	16 (18)	**52 (58)**	18 (20)	3 (3)	0 (0)	9 (10)	18 (20)	**41 (46)**	19 (21)	2 (2)
Physicians (n = 67)	12 (18)	**45 (67)**	10 (15)	0 (0)	**46 (69)**	19 (28)	2 (3)	0 (0)	0 (0)	13 (19)	**50 (75)**	4 (6)	0 (0)	0 (0)

***NA*** *: not assessed; **NAP**: not applicable.*
